# Semantic Intrusion Errors Overcome the Impact of Literacy and Educational Attainment in Black/African Americans with and without Mild Cognitive Impairment

**DOI:** 10.4236/aad.2025.144008

**Published:** 2025-11-10

**Authors:** Alexandra Ortega, Kirsten Horne Crenshaw, Triana Abel, Carlos Valentín-Camuñas, Stephanie Remedios, Brooke Bosworth, Sofia Ramirez, Dylan Hinton, Aleksandra Nedza, Elizabeth A. Crocco, Rosie E. Curiel-Cid, David Loewenstein

**Affiliations:** 1 Florida Alzheimer’s Disease Research Center (ADRC), Miami, USA; 2 Center for Cognitive Neuroscience and Aging (CNSA), Department of Psychiatry and Behavioral Sciences, Miller School of Medicine, University of Miami, Miami, USA

**Keywords:** Mild Cognitive Impairment, Semantic Intrusions, Proactive Interference, Literacy, African Americans

## Abstract

The Loewenstein-Acevedo Scale for Semantic Interference and Learning (LASSI-L) is a novel test of learning and inability to recover from proactive interference. It has been successfully employed with older adults at risk for cognitive impairment but the association between literacy, educational achievement, and LASSI-L performance in underserved Black/African American (B/AA) older adults remains unknown. 83 participants were evaluated as part of a longitudinal observational study to render a consensus diagnosis based on comprehensive clinical history and neuropsychological evaluation. Participants were classified into two groups: 43 were diagnosed with amnestic mild cognitive impairment (aMCI) and 40 were cognitively unimpaired (CU). The Wide Range Achievement Test-IV (WRAT-IV) was used to determine levels of literacy. Participants completed the LASSI-L, an AD sensitive cognitive challenge test which was not utilized for diagnosis to avoid circularity. The CU group outperformed the aMCI group across all LASSI-L measures after demographic covariate adjustment. Robust associations were found between education, literacy, and correct responses on different LASSI-L word lists (but not semantic intrusion errors). In contrast, aMCI participants largely exhibited no associations between LASSI-L indices and literacy effects. Stepwise logistic regression achieved an overall accuracy of 81% in classifying cognitive status (84% CU specificity, 71% aMCI sensitivity). Literacy was related to certain LASSI-L measures of learning in CU participants, but not among individuals with aMCI. Even when adjusting for demographic covariates, the LASSI-L effectively distinguished B/AA CU and aMCI groups. Results underscore the practicality of the LASSI-L to assess cognitive impairment in individuals with lower literacy proficiency, as our model correctly distinguish 84% of cognitively unimpaired individuals and 71% of individuals with amnestic mild cognitive impairment.

## 1. Introduction

Numerous studies have demonstrated how reading ability, health literacy, and educational attainment influence cognitive performance on neuropsychological testing in Black/African Americans (B/AA) [1]-[7]. For example, higher levels of literacy and educational attainment have been linked to a delayed onset of mild cognitive impairment (MCI) and slower cognitive decline, likely due to their hypothesized role in cognitive reserve [7]-[11]. Both years of formal education and literacy-related skills contribute to cognitive reserve; however, literacy may be a more proximal indicator than years of schooling, as it has been shown to predict cognitive decline more accurately and associated with brain structural integrity in midlife [12] [13]. However, while commonly used cognitive assessments for Alzheimer’s disease (AD) have demonstrated effectiveness in detecting cognitive decline, their validity may be limited for individuals with lower levels of education and literacy due to cultural and racial biases in cognitive testing and normative data [14].

Assessing cognitive impairment in ethnically diverse individuals with limited literacy and educational backgrounds presents significant challenges [6] [14]-[16]. Many neuropsychological tests were developed and normed on highly educated individuals and of Caucasian descent, which raises concerns regarding their applicability to ethnically differing populations and more so with lower educational attainment [17]-[19]. Also, clinicians may struggle to differentiate between true cognitive deficits and the effects of limited literacy, leading to potential misclassification of cognitive status [6]. This issue is particularly pronounced among historically underserved populations, such as Black/African American (B/AA) older adults, where cognitive assessments may not be culturally or educationally appropriate, increasing the risk of false-positive diagnoses [20]-[22]. Indeed, evidence suggests that illiterate individuals tend to score significantly lower on dementia screening tools, further highlighting the need for literacy-sensitive assessments [4].

Research also shows that the completion of formal education alone does not necessarily ensure a comparable level of literacy across diverse demographic groups, due to factors such as school quality and associated contextual and socioeconomic factors [23] [24]. As a result, researchers have suggested incorporating literacy measures, such as the Word Reading subtest of the Wide-Range Achievement Test-4th Edition (WRAT-IV) to provide a more accurate estimation of premorbid functioning and cognitive reserve [25] [26]. This approach is especially critical in communities with lower educational attainment, where traditional years-of-education metrics may not fully capture cognitive resilience and abilities. Recent efforts have focused on increasing the inclusion of B/AA older adults in AD and Alzheimer’s Disease-Related Dementias (ADRD) research. Promising cognitive measures, such as the Loewenstein-Acevedo Scales for Semantic Interference and Learning (LASSI-L), have demonstrated utility in differentiating Hispanic/Latino and B/AA individuals with amnestic mild cognitive impairment (aMCI) from those who are cognitively unimpaired (CU) [27]-[29]. However, limited research has examined the influence of education and literacy on specific LASSI-L indices.

In this study, we hypothesize that among B/AA older adults, literacy (as measured by the WRAT-IV Word Reading subtest score) will be more strongly associated with LASSI-L performance in CU individuals than in those with aMCI. Furthermore, we propose that the LASSI-L will effectively distinguish between CU and aMCI participants, even after controlling demographic variables such as education and literacy. Lastly, we anticipate that specific LASSI-L indices, particularly those related to proactive semantic interference and learning (e.g., initial Cued A2 learning and Cued B1 intrusion errors), will significantly contribute to the classification of cognitive status among B/AA older adults, yielding high specificity and sensitivity.

## 2. Methods

### 2.1. Participants

A total of 83 individuals who self-identified as Black or African American (B/AA) were recruited from an NIA-funded R01 participant pool of community-dwelling older adults. The NIA adheres to the Office of Management and Budget (OMB) standards for racial and ethnic classifications, which define the B/AA category as: “A person having origins in any of the Black racial groups of Africa.” Participants were recruited through various community engagement events across Miami-Dade and Broward counties, including churches, faith-based organizations, community centers, health clinics, senior activity centers, and government institutions. The overall sample had a mean age of 63.8 years (SD = 5.6) and an average educational attainment of 13.3 years (SD = 2.6). The sample was 49.2% female. Participation was voluntary and participants were modestly compensated. All individuals underwent a comprehensive annual clinical and neuropsychological diagnostic battery. This study, conducted at the University of Miami Miller School of Medicine, is IRB-approved.

### 2.2. Baseline Diagnostic Criteria

Baseline clinical diagnoses were determined using the following procedure: a skilled and experienced clinician, unaware of the neuropsychological test results, administered a standardized clinical assessment that included the Clinical Dementia Rating Scale [30], participant and informant interviews to assess memory and other cognitive and functional issues. All participants were community-dwelling, capable of performing daily activities independently, had knowledgeable collateral informants, and did not meet DSM-5-TR criteria for Major Neurocognitive Disorder, active Mood or Psychotic Disorder, or any other significant neuropsychiatric condition [31] that would interfere with reliable assessment. Participants showing clear signs of cognitive decline were assigned a CDR score of 0.5. After the clinical evaluation, a trained psychometrician administered a standardized neuropsychological assessment, including the LASSI-L. To avoid criterion contamination, performance on the LASSI-L was excluded from the diagnostic process.

The neuropsychological battery included a range of assessments from the National Alzheimer’s Coordinating Center’s (NACC) Uniform Data Set (UDS) [32]. Tests covered various domains, such as global functioning (Mini-Mental State Examination [MMSE]); memory (Hopkins Verbal Learning Test-Revised [HVLT-R], NACC story passages, Benson Figure Delayed Recall); language (Controlled Oral Word Association Test, Category Fluency); processing speed (Trail Making Test Part A); and executive functioning (Trail Making Test Part B, Stroop Test). To assess literacy levels, the Wide Range Achievement Test (WRAT-IV) was administered to all participants [33]. When applicable, we used age-appropriate normative data developed and validated for use with B/AA older adults, titled the Mayo’s Older African Americans Normative Studies (MOAANS) on commonly used neuropsychological tests [32]-[34].

### 2.3. Amnestic Mild Cognitive Impairment Group (aMCI; n = 43)

Based on the clinical interview, and outcomes of the neuropsychological assessments, individuals were categorized as having amnestic mild cognitive impairment (aMCI) if all of these specific criteria were met: a) significant memory complaints by either the participant or collateral informant; b) substantiated evidence of memory and/or other cognitive decline through clinical evaluation and medical history; c) a Global CDR score of 0.5; and d) one or more memory or non-memory measures described above at least 1.5 standard deviations or more below the expected normative data for B/AA older adults.

### 2.4. Cognitively Unimpaired Group (CU; n = 40)

Based on the independent clinical interview and results from the neuropsychological assessments, an individual was categorized as cognitively unimpaired (CU) if the subsequent criteria were satisfied: a) the absence of any memory complaints by the participant and/or collateral informant; b) no indication of memory and/or other cognitive decline based on clinical evaluation or medical history; c) a Global CDR score of 0; and d) all memory or non-memory cognitive tests falling less than 1.0 standard deviation below the age and education-adjusted normative data for B/AA older adults.

### 2.5. Loewenstein-Acevedo Scales for Semantic Interference and Learning (LASSI-L)

**Figure 1.**
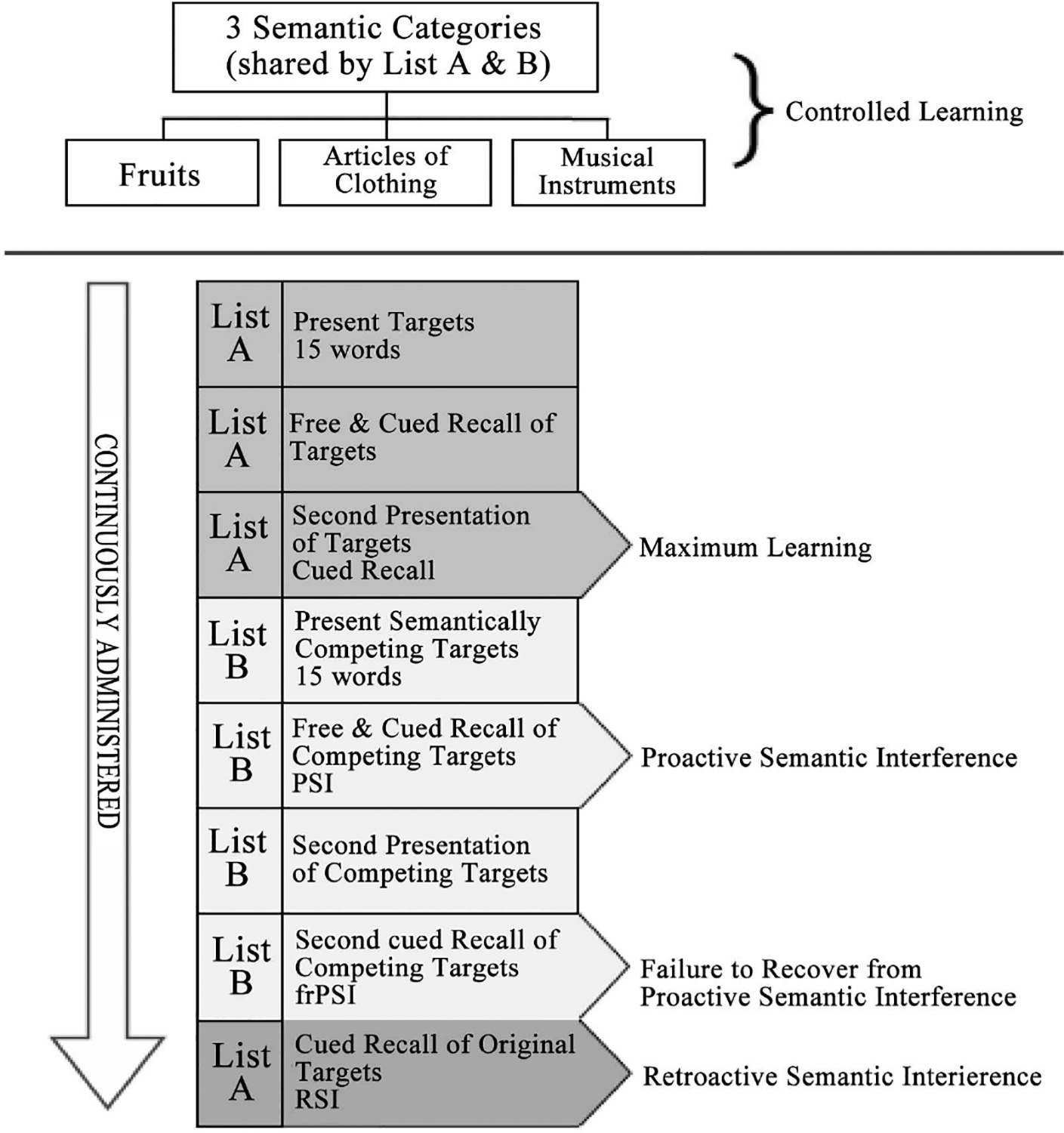
LASSI-L flowchart breakdown.

The LASSI-L utilizes a controlled learning paradigm designed to optimize the encoding of target words organized into three distinct semantic categories [35]: fruits, clothing, and musical instruments, see Figure 1 below for a visual flow chart summary of the LASSI-L test paradigm. Participants are initially presented with a list of words, which are both verbally and visually presented to enhance learning and create stronger memory traces. After this, a second list of semantically similar words is introduced, increasing task complexity and allowing for the observation of how individuals manage interference between the two lists. Some participants may experience semantic intrusion errors (SIE), where they mistakenly mix words from the first list with those from the second. These errors arise because the brain struggles to inhibit previously learned information, a process known as proactive semantic interference (PSI), where older memories interfere with the ability to learn new information [36]. The LASSI-L then evaluates participants’ ability to recover from PSI, a process known as failure to recover from proactive semantic interference (frPSI). FrPSI refers to continued difficulties with PSI despite multiple attempts to present the new targets for learning [37].

Measures, of frPSI in particular have proven to be powerful in distinguishing individuals with aMCI from CU older adults [36] [38], and they show strong correlations with neurodegeneration in brain regions prone to AD [39]. Intrusion errors observed in frPSI are sensitive to the effects of amyloid load and reflect deficits in source memory, self-monitoring, and inhibitory control [40] [41]. These cognitive markers are also relevant for detecting aMCI and correlate with various biomarkers of AD and neurodegeneration, highlighting their importance in cognitive assessment, particularly among culturally diverse older adults [37] [42]-[46]. As previously noted, LASSI-L was not used for diagnostic purposes in this study to avoid potential circularity. See Table 1 below for participant demographics and LASSI-L variables, and Table 2 for LASSI-L variables for cued recall and intrusions for both CU and aMCI groups.

## 3. Results

The mean age was almost identical for both groups, with the CU group averaging 63.7 years (SD = 4.8) and the aMCI group averaging 63.8 years (SD = 6.3). However, the CU group had a significantly higher mean educational attainment (M = 13.93 years, SD = 2.6) compared to the aMCI group (M = 12.70 years, SD = 2.4; F = 5.049, p = 0.027), with a small to moderate effect size (Eta Squared = 0.059). Regarding sex, the CU group has a higher percentage of females (58.1%) compared to the aMCI group (37.5%), but this difference is not statistically significant (F = 2.76, p = 0.097). The CU group also scored significantly higher on the WRAT-IV Reading test than the aMCI group (CU: M = 57.12, SD = 10.1; aMCI: M = 49.43, SD = 9.6; F = 13.845, p < 0.001) with a moderate effect size (Eta Squared = 0.148). Similarly, the CU group outperformed the aMCI group on the MMSE (CU: M = 28.44, SD = 1.40; aMCI: M = 26.15, SD = 1.91), with a large effect size (Eta Squared = 0.327) and a statistically significant difference (F = 39.413, p < 0.001).

Among CU individuals, a strong and statistically significant positive correlation was observed between years of formal education and literacy levels (r = 0.724, p < 0.001). Further, both education and literacy were positively correlated with performance on all cued recall tasks, including Cued A2 (r = 0.490, p < 0.001), Cued B1 (r = 0.437, p < 0.01), and Cued B2 (r = 0.415, p < 0.01). These results suggest that CU individuals with higher levels of education and greater literacy abilities tended to perform better on memory tasks that require accurate recall in response to semantic cues. It should be noted however, that neither education (Cued B1 r = −0.038, p > 0.05; Cued B2 r = −0.253, p > 0.05), nor literacy (Cued B1 r = −0.247, p > 0.05; Cued B2 r = −0.283, p > 0.05) were significantly associated with the number of semantic intrusions in the CU group, indicating that these factors may not influence the tendency to produce erroneous responses during recall (See Table 3 below for CU group results; See Table 4 for aMCI group results). Among individuals with aMCI, the relationship between formal education and literacy was noticeably weaker compared to the CU group. While a significant correlation was still present in the aMCI group, it was quite modest in strength (r = 0.312, p < 0.05). Additionally, unlike in the CU group, neither formal education nor literacy showed significant associations with any of the LASSI-L indices. These findings suggest that, within the aMCI group, the influence of educational attainment and literacy on memory performance is less pronounced, potentially reflecting the complex interplay of cognitive impairment and educational background in shaping test performance.

Performance on the LASSI-L also differed notably between groups. CU participants outperformed those with aMCI on all Cued Recall tasks and exhibited fewer semantic intrusion errors, with all comparisons reaching a high level of statistical significance (p < 0.001). To account for initial differences in education, MMSE, and WRAT-IV scores between groups, these variables were included as covariates in ANCOVA models. Results confirmed that CU participants continued to show superior performance on all LASSI-L Cued Recall tasks (Cued A2, B1, B2) and had fewer semantic intrusions, reinforcing the robustness of these cognitive differences even after statistical adjustment for potential confounders. Stepwise logistic regression was employed to determine those LASSI-L variables that were best distinguished between diagnostic groups. Two key LASSI-L measures—initial Cued A2 learning and Cued B1 intrusion errors—as significant predictors distinguishing CU from aMCI participants. The resulting model demonstrated strong classification accuracy, correctly identifying 80.7% of cases. More specifically, it achieved a specificity of 83.7 %, meaning it accurately classified CU individuals, while its sensitivity—its ability to correctly identify those with aMCI—was 71.1% (See Table 5 below). These findings underscore the utility of LASSI-L measures in distinguishing cognitive status and highlight their potential role in detecting early memory impairment in AA adults and distinguishing between CU and aMCI.

## 4. Discussion

The current research undertook a thorough investigation into the intricate connections among educational attainment, literacy, and cognitive performance on the novel cognitive challenge task, the LASSI-L, within a B/AA cohort of CU individuals and their counterparts with aMCI. All indices of the LASSI-L showed robust differences between groups. Further, covariate analyses indicated significant LASSI-L differences between aMCI and CU groups after adjustment for education, literacy, and MMSE scores. Preliminary research on a smaller sample by [29] demonstrated the effectiveness of LASSI-L in distinguishing between B/AA aMCI and CU individuals, was most highly related to the ratio of intrusion errors on Cued List B1. We obtained consistent results with our larger cohort in this larger investigation. However, initial learning of the original targets in conjunction with List B provided the most discriminatory power for distinguishing B/AA CU and aMCI participants suggesting that initial learning strength may have an important role in aMCI among this larger cohort. More importantly, the current investigation delves into the important question regarding the nuanced relationships between education, literacy, and LASSI-L measures within CU and aMCI groups, contributing a novel perspective to the existing literature.

Our study aligns with literature suggesting a reduction of the association between education and cognitive performance in the presence of increased cognitive impairment in non-AA populations [6] [14]-[16]. Whereas there was a strong association between years of formal education and literacy among B/AA CU participants as well as higher literacy and correct scores on different learning trials, this did not hold true for those B/AA participants diagnosed with aMCI. Further, among both CU and aMCI B/AA groups, there was no relationship between semantic intrusions on the second list on either the first administration or the second administration. Semantic intrusions are thought to represent more executive dysfunction in that the person must self-monitor and not include targets from the first list into the second list [47] [48]. The absence of a significant relationship in the context of semantic intrusions suggests that executive brain-based factors may be the drivers of these types of errors beyond education or literacy in this population [40].

The current study has several notable strengths. First, the B/AA participant group underwent a thorough clinical and neuropsychological evaluation conducted by experts with specific expertise in diagnosing cognitive impairment within this population. Importantly, the neuropsychological assessments incorporated culturally and ethnically appropriate normative data, enhancing diagnostic accuracy and relevance for AA participants. Second, to ensure methodological rigor and avoid issues of circularity, the LASSI-L was excluded from the diagnostic decision-making process used to classify participants as aMCI or CU. This strategy minimizes the risk of tautology, where a test used in diagnosis is later used as an outcome or predictor. Additionally, the study employed the Wide Range Achievement Test, Fourth Edition (WRAT-4), as a literacy measure for AA participants [5] and took great care in evaluating participants’ educational backgrounds. Finally, this research incorporated a novel cognitive stress test that has shown increasing utility in studies involving both Hispanic and B/AA populations [27]-[29].

The LASSI-L emerges as a valuable tool for assessing cognitive impairment in diverse populations, contributing to the goal of reducing disparities in cognitive healthcare. Further research is warranted to validate and refine the utility of LASSI-L subtests in clinical settings and explore additional factors contributing to cognitive disparities among older adults. It is imperative to acknowledge the potential weaknesses of the present study to ensure a nuanced interpretation of the findings. We studied 83 participants, with a minimum of 40 participants per cell. Enhancing the generalizability of the findings would benefit from a larger participant pool, allowing for a more robust analysis and a better representation of the diverse characteristics within the population. In addition, while the study concentrated on well-characterized amnestic aMCI and CU B/AA individuals, the generalizability of the results may be influenced by the specific characteristics of the sample. It is important to recognize that aMCI can stem from various conditions, including AD, cerebrovascular disease, renal failure, diabetes, and other medical co-morbidities that can potentially attenuate group differences in cognitive testing due to underlying pathological differences. For instance, the relationship between semantic intrusion errors and enhanced amyloid burden in individuals with aMCI suggests potential variability in the etiology of cognitive impairment. The admixture of underlying etiologies among cohorts may in part explain some of the differences obtained in [29] and this present larger study.

Given the community-based recruitment approach, the base rates of underlying AD may be lower compared to settings where individuals are presenting with explicit memory complaints, impacting the external validity of the findings. Future investigations may benefit from not delving into advanced diagnostic measures such as brain amyloid PET, brain MRI, p-tau 217 plasma biomarkers, or comprehensive blood panels including Hemoglobin A1C, Bun-Creatinine, lipids, and other metabolic markers. Integrating these advanced diagnostic measures in future research could provide valuable insights into the effects of underlying etiologies or co-pathologies on cognitive test performance. In summary, this study underscores the intricate relationship between education, literacy, and cognitive performance among B/AA older adults. While both literacy and educational attainment significantly influence cognitive outcomes in CU individuals, this effect appears diminished once individuals progress to the aMCI stage, particularly in relation to LASSI-L performance. The observed disparities between CU and aMCI groups highlight the urgent need for culturally responsive and literacy-informed assessment tools, especially when working with historically underserved populations. Findings from this study support the LASSI-L as a promising measure for detecting subtle cognitive changes in B/AA older adults and other diverse aging populations, reinforcing its potential to advance equity in cognitive assessment. Continued research is essential to further validate these subtests and to identify additional sociocultural and contextual factors that contribute to disparities in cognitive aging. Results also suggest the potential of cognitive challenge tests for assessing B/AA older adults with low educational attainment. By refining diagnostic tools and practices, we move closer to ensuring more accurate, fair, and inclusive cognitive health evaluations for all older adults.

## Figures and Tables

**Table 1.** Demographic and LASSI-L variables for older African American Cognitively Unimpaired (CU) and Amnestic MCI (aMCI) older adults.

	B/AA CU Participants (n = 43)	B/AA aMCI Participants (N = 40)	F-Value	P-Value	Eta Squared (Effect Size)
Age (range 52 – 84)	63.70 (SD = 4.8)	63.78 (SD = 6.3)	0.004	0.950	0.000
Educational Attainment (range 8 – 20)	13.93 (SD = 2.6)	12.70 (SD = 2.4)	5.049	0.027	0.059
Sex (% Female)	58.1%	37.5%	2.76	0.097	NA
MMSE (range 23 – 30)	28.44 (SD = 1.40)	26.15 (SD = 1.91)	39.413	<0.001	0.327
WRAT-IV Reading (24 – 70)	57.12 (SD = 10.1)	49.43 (SD = 9.6)	13.845	<0.001	0.148
LASSI-L Cued Recall A2 (range = 6 – 15)	13.19 (SD = 1.7)	10.65 (SD = 1.8)	42.235	<0.001	0.343
LASSI-L Cued Recall B1 (range = 0 – 12)	7.67 (SD = 2.6)	5.20 (SD = 2.2)	21.067	<0.001	0.206
LASSI-L Cued Recall B2 (range = 0 – 15)	11.14 (SD = 2.4)	8.08 (SD = 2.7)	29.859	<0.001	0.269
LASSI-L Cued Recall B1 Intrusions (0 – 13)	2.35 (SD = 1.9)	4.93 (SD = 3.1)	20.685	<0.001	0.203
LASSI-L Cued Recall B2 Intrusions 0 – 13)	1.72 (SD = 1.5)	3.88 (SD = 2.6)	21.825	<0.001	0.212

Note: 2 × 2 Chi-square analyses for sex employed Yate’s correction for discontinuity.

**Table 2.** LASSI-L variables for recall and intrusions older African American Cognitively Unimpaired (CU) and Amnestic MCI (aMCI) older adults.

	AA CU Participants (n = 43)	AA aMCI Participants (N = 40)	F-Value Corrected for WRAT-IV Reading, Education, and MMSE	P-Value	Partial Eta Squared (Effect Size)
LASSI-L Cued Recall A2 (range = 6 – 15)	12.58 (SE = 0.27)	11.265 (SE = 0.27)	9.880	<0.001	0.114
LASSI-L Cued Recall B1 (range = 0 – 12)	7.11 (SE = 0.39)	5.86 (SE = 0.41)	4.009	0.049	0.049
LASSI-L Cued Recall B2 (range = 0 – 15)	10.33 (SE = 0.40)	8.93 (SE = 0.41)	4.978	0.029	0.061
LASSI-L Cued Recall B1 Intrusions (0 – 13)	2.00 (SE = 0.36)	3.857 (SE = 0.37)	14.185	<0.001	0.155
LASSI-L Cued Recall B2 Intrusions 0 – 13)	1.72 (SD = 1.5)	3.88 (SD = 2.6)	7.61	0.007	0.090
Cued B1 Intrusions/Total Responses (range = 0 to 1.0)	0.264 (SE = 0.03)	0.426 (SE = 0.03)	10.597	0.002	0.121
Cued B2 Intrusions/Total Responses range = (0 – 0.79)	0.194 (SE = 0.03)	0.331 (SE = 0.03)	12.186	<0.001	0.137

Note: Means and F-values adjusted for education, MMSE scores and WRAT-IV Reading Literacy is presented.

**Table 3.** The relationship between education and literacy level among Cognitively Unimpaired (CU) Black/African American older adults.

	Education (years)	Literacy (WRAT-IV Reading)	Cued A2	Cued B1	Cued B2	Cued B1 Intrusions	Cued B2 Intrusions
Education (years)	NA	r = 0.724***	r = 0.490***	r = 0.437**	r = 0.415**	r = −0.038	r = −0.253
Literacy (WRAT-IV Reading)	r = 0.724***	NA	r = 0.511***	r = 0.484***	r = 0.584**	r = −0.247	r = −0.283

Note:

* = p ≤ 0.05,

** = p ≤ 0.01;

*** = p ≤ 0.001.

After correction for False Discovery Rate (FDR), all statistically significant results remained statistically significant at p ≤ 0.05.

**Table 4.** The relationship between education and literacy among Black/African American Older adults with Amnestic Mild Cognitive Impairment (aMCI).

	Education (years)	Literacy (WRAT-IV Reading)	Cued A2	Cued B1	Cued B2	Cued B1 Intrusions	Cued B2 Intrusions
Education (years)	NA	r = 0.312*	r = 0.280	r = −0.032	r = 0.031	r = −0.165	r = −0.158
Literacy (WRAT-IV Reading)	r = 0.312*	NA	r = 0.170	r = 0.048	r = 0.204	r = −0.058	r = −0.170

Note:

* = p < 0.05. After correction for False Discovery Rate (FDR), no correlations were statistically significant.

**Table 5.** Stepwise regression using LASSI-L subtests as predictors of Cognitive Status (CU group versus aMCI group).

Equation Variables							
		B	S.E.	Wald	df	Sig.	Exp (B)
Step 1^a^	LCUEDA2	−0.792	0.177	20.098	1	<0.001	0.453
	Constant	9.466	2.162	19.176	1	<0.001	12917.402
Step 2^b^	LCUEDA2	−0.698	0.181	14.911	1	<0.001	0.498
	LCuedB1INT	0.290	0.118	6.033	1	0.014	1.337
	Constant	7.241	2.262	10.252	1	0.001	1395.957

Note:

a = Variable(s) entered on step 1: LCUEDA2;

b = Variable(s) entered on step 2: LCuedB1INT.

Overall Classification = 80.7% Specificity = 83.7%; Sensitivity = 71.1%.
